# Epidemiology of Undifferentiated Carcinomas

**DOI:** 10.3390/cancers14235819

**Published:** 2022-11-25

**Authors:** Matthew G. K. Benesch, Shalana B. L. O’Brien

**Affiliations:** Department of Surgical Oncology, Roswell Park Comprehensive Cancer Center, Buffalo, NY 14263, USA

**Keywords:** dedifferentiated, anaplastic, pleomorphic, histopathology

## Abstract

**Simple Summary:**

When compared microscopically to normal cells, cancer cells are graded on a continuum from well differentiated to undifferentiated, meaning that well differentiated cancer cells look similar to normal cells and undifferentiated ones look very different. Patient survival outcomes typically decrease along this continuum. However, some cancer cells are so undifferentiated that it is not certain from which cell type they arose from. It is not known if these patients have worse outcomes compared to undifferentiated cancers where the cell type origin can be determined. In this study, we show that patient outcomes from undifferentiated cancers depend on the site, and generally, outcomes are worse compared to undifferentiated cancers with a discernible cell type, but there are some notable exceptions. This work illustrates that tumor site has significant impacts on patient survival even when accounting for multiple demographic, clinical, and histological factors.

**Abstract:**

Undifferentiated carcinomas are rare cancers that lack differentiation, such that they cannot be classified into any conventional histological subtype. These cancers are uniquely codified and are contrasted to carcinomas with an ascertained histology that are grade classified as poorly differentiated, undifferentiated, or anaplastic. Given their rarity, there are no standardized overviews of undifferentiated carcinomas in the literature, and it is unknown if their classification indicates a unique prognosis profile. In this study, we summarize the clinicodemographic and mortality outcomes of undifferentiated carcinomas in twelve primary sites and for unknown primaries, comprising 92.8% of all undifferentiated carcinomas diagnosed from 1975–2017 in the Surveillance, Epidemiology, and End Results Program (SEER). Incidence has decreased to 4 per 1 million cancer diagnoses since 1980. Relative to the most common undifferentiated cancers with a defined histology, undifferentiated carcinomas have overall worse prognosis, except in nasopharyngeal and salivary gland cancers (hazard ratio (HR) 0.7–1.3). After correction for age, sex, race, detection stage, and treatment (surgery, chemotherapy, and radiotherapy), the mortality HR averages 1.3–1.4 for these cancers relative to histologically ascertainable undifferentiated cancers. However, there is a wide variance depending on site, signifying that survival outcomes for undifferentiated carcinomas depend on factors related to site tumor biology.

## 1. Introduction

Undifferentiated carcinomas are formally defined by the World Health Organization (WHO) as malignant epithelial neoplasms which lack evidence of glandular, squamous, or urothelial cell differentiation [1]. Differentiation refers to how histologically similar a neoplasm resembles normal tissues. In general, neoplasms are pathologically graded on a scale from well to undifferentiated, and along this continuum, disease tends be become increasingly aggressive with worsened outcomes. To be classified as an undifferentiated carcinoma, the neoplasm cannot have distinguishable morphological or immunophenotypical features that would fit a specific carcinoma category, such as adenocarcinomas or squamous cell carcinomas [2,3]. These cancers could arise de novo or along a dedifferentiation process from a differentiated carcinoma [3]. In some circumstances, a cancer at a primary site might have a differentiated phenotype but become undifferentiated at a metastatic site [4]. This can create a scenario where a tumor biopsy at a distant site returns an undifferentiated histotype, and in the absence of either a primary site lesion or an identifiable immunostaining pattern, this is referred to as a cancer of unknown primary [5]. Prognostication and treatment of these cancers is heterogenous, but the outcome is usually poor [5].

For most tumor sites, differentiation for histologically definable cancers has historically been scored on a four-point scale: well, moderate, poor, and undifferentiated/anaplastic. Patient survival tends to worsen along this differentiation pathway, apart from the poor and undifferentiated categories, where survival after controlling for other aspects of tumor biology is statistically similar for these two later groupings [6,7]. This is one of the factors that lead to the grouping of “poor” and “undifferentiated” differentiation into a single category for most cancer sites in the 8th edition of the American Joint Committee on Cancer (AJCC) Staging Manual in 2018 [8].

Given the rarity of undifferentiated carcinomas, there is a paucity of information on their epidemiology. It is also unknown if the inability to assign a histological phenotype for carcinomas is an independent predictor of patient survival when compared to undifferentiated tumors of ascertained histologies, and if this is consistent across all tumor sites. To robustly examine the epidemiology of undifferentiated carcinomas, we used the Surveillance, Epidemiology, and End Results (SEER) database in this study. The SEER database is a population-based cancer registry with over 40 years of near universal capture of cancer patients for about one-third of the population of the United States [9,10]. As an invaluable resource for quantifying data on histopathology with survival outcomes, we provide the most extensive examination to date of the epidemiology of undifferentiated carcinomas via a site-stratified analysis for these rare and understudied cancers.

## 2. Materials and Methods

### 2.1. Patient Selection

The National Cancer Institute’s SEER database comprised from 17 SEER cancer registries was employed using data from 1975–2019 as previously described [6,7]. Patients diagnosed through to 2017 were included to ensure at least two years of survival data was present for all patients. Data release from the SEER database does not require informed patient consent or review by institutional review board. The SEER database was accessed and searched in compliance with signed user agreements. A complete outline of exclusion criteria and its effects on case counts is presented in Table S1. Definitions of variables analyzed are presented in Table S2. We limited our analysis to sites with at least 200 cases of undifferentiated carcinomas (Table S3). We then compared demographic and outcomes data for undifferentiated carcinomas against the top one or two definable undifferentiated histologies for each the selected sites. The resulting ICD-O-3 codes used for patient selection are detailed in Table S2.

### 2.2. Statistical Analysis

All selected data from SEER cancer registries was imported into Stata 15.1 (StataCorp LLC, College Station, TX, USA) for statistical analysis following case listing downloading using SEER*Stat 8.4.1 (Surveillance Research Program, National Cancer Institute, Calverton, MD, USA). A complete case analysis was completed after variable definition (Tables S1 and S2).

Baseline patient characteristics were compared with the *t* and *χ*^2^ tests for continuous and categorical variables, respectively. Univariate and multivariable Cox proportional hazard regression was used to determine the association of mortality with cancer histology type, adjusting for age, sex, race, detection stage, grade differentiation, surgery, radiotherapy, and chemotherapy. All hazard ratios were calculated with 95% confidence intervals. Use of surgery, radiotherapy, and chemotherapy as treatment variables were binary. All *p*-values were two-sided, with a threshold of 0.05 to determine statistical significance. Survival curves were plotted using the Kaplan–Meier method, with *p*-values for survival curves generated by the log rank test. Graphs were plotted using Origin Pro 2022 (OriginLab Corporation, Northampton, MA, USA). Using SEER 18 (2000–2018) data with SEER*Stat 8.4.1, yearly incidence and mortality rates were calculated and age-adjusted to the 2000 United States standard population with the age variable recode <1-year-olds. Cause-specific survival and relative survival were calculated with the same dataset and software, and were both age standardized to the International Cancer Survival Standard 1-Age 15+ variable via the actuarial method, and Ederer II cumulative expected method for relative survival.

## 3. Results

### 3.1. Distribution and Survival Curves for Undifferentiated Carcinomas by Site

**Figure 1:**
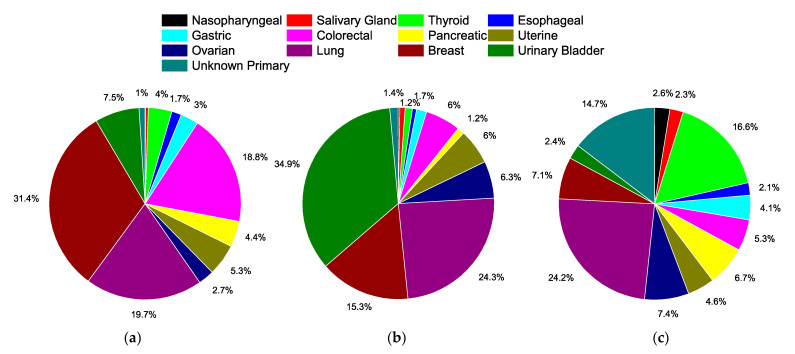
Distribution of cancers by site analyzed in SEER, 1975–2017. (**a**) All cases by sites examined, totaling 5.1 million cases. (**b**) All undifferentiated cases with defined histological subtype by site examined, totaling 252,930 cases. (**c**) All undifferentiated carcinomas by site examined totaling 11,292 cases. Data labels represent percentages, and markers are omitted if less than 1%.

Figure 1 presents the distribution of all anatomical sites included for analyses, encompassing 92.8% of all undifferentiated carcinoma cases (Table S3). Among these sites, breast (31.4%), lung (19.7%), and colorectal (18.8%) are the most common cancers overall (Figure 1a), but for undifferentiated cases with a defined histology, the top three sites are urinary bladder (34.9%), lung (24.3%), and breast (15.3%) (Figure 1b). For undifferentiated carcinomas, the top three sites are lung (24.2%), thyroid (16.6%), and unknown primary (14.7%) (Figure 1c).

**Figure 2:**
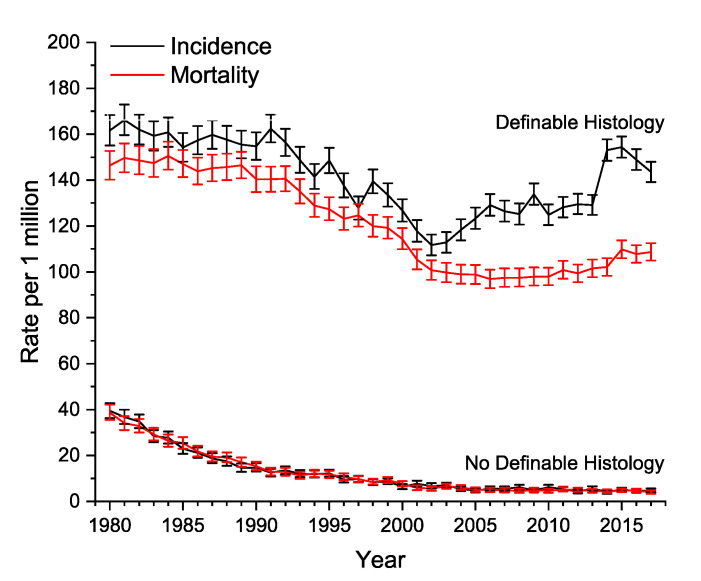
Age-adjusted yearly incidence and mortality rates for undifferentiated carcinomas. Definable histology refers to undifferentiated carcinomas with an ascribed histology, and no definable histology refers to undifferentiated carcinomas without an ascribed histology. Error bars represent 95% confidence intervals.

Figure 2 illustrates age-adjusted incidence and mortality curves for both histologically definable and undefinable undifferentiated carcinomas. For the undefined undifferentiated carcinomas, the two curves are in near perfect overlap, and have decreased nearly 10-fold since 1980. For the definable carcinomas, there has been a slight decrease in incidence over time, with an even greater rate of decrease in mortality after 2002.

**Figure 3:**
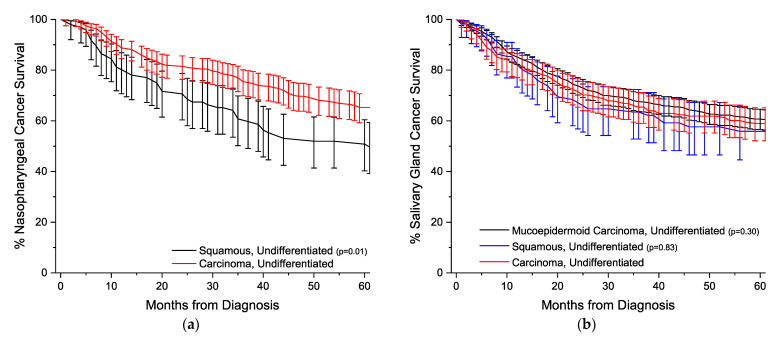

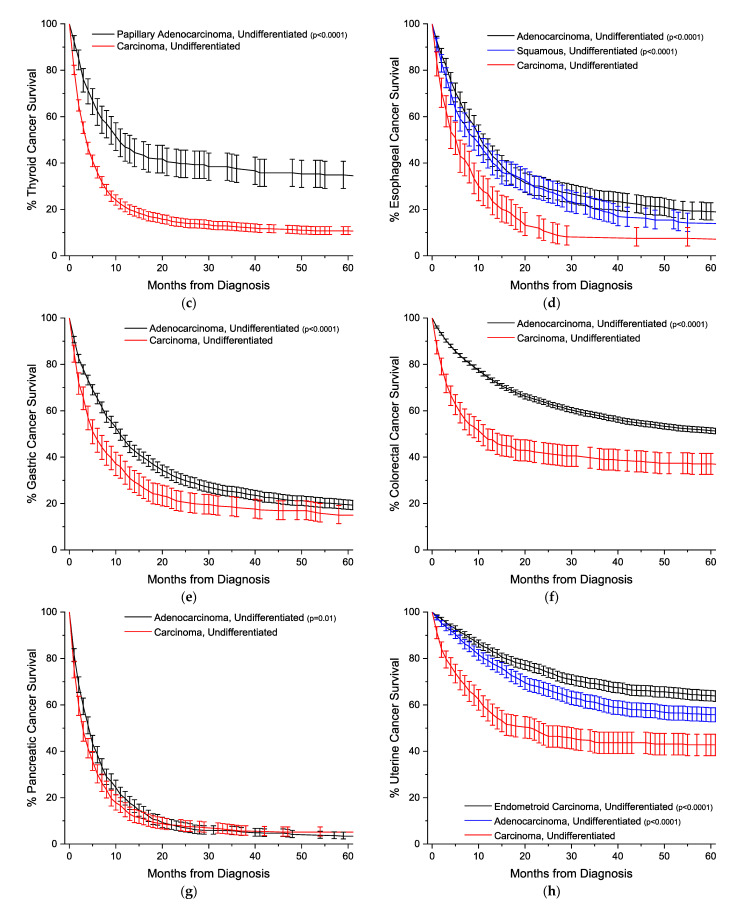

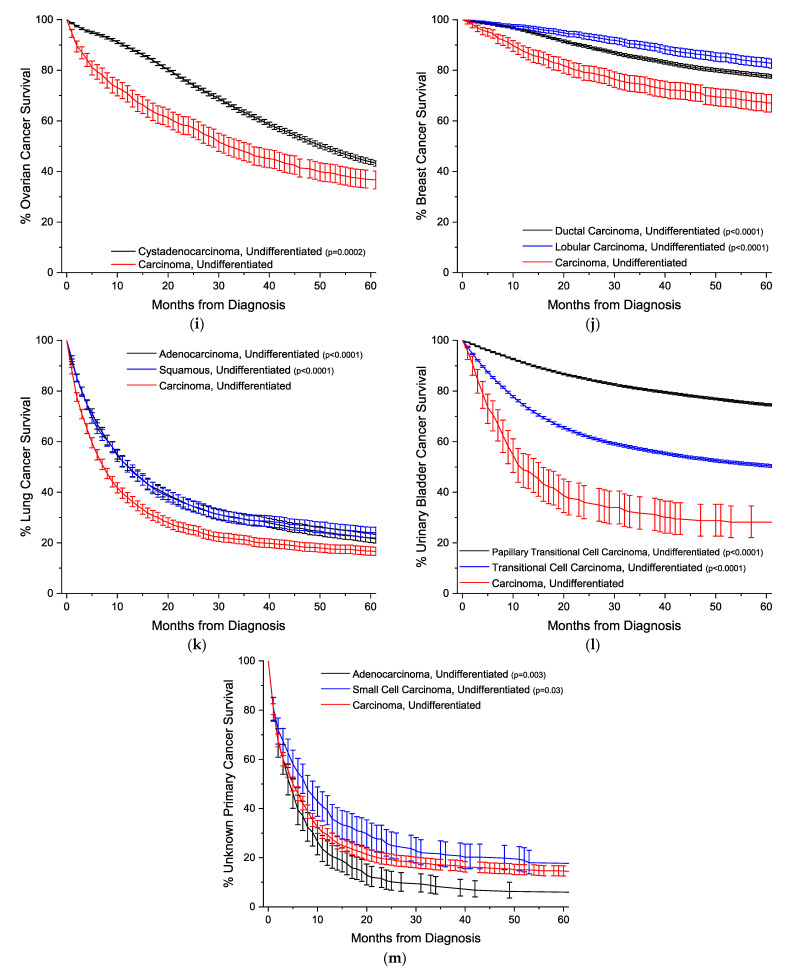
Kaplan–Meier overall survival curves. All survivor functions are shown with 95% confidence intervals. (**a**) Nasopharyngeal cancer. (**b**) Salivary gland cancer. (**c**) Thyroid cancer. (**d**) Esophageal cancer. (**e**) Gastric cancer. (**f**) Colorectal cancer. (**g**) Pancreatic cancer. (**h**) Uterine cancer. (**i**) Ovarian cancer. (**j**) Breast cancer. (**k**) Lung cancer. (**l**) Urinary bladder cancer. (**m**) Unknown primary cancer. All *p*-values are relative to the Carcinoma, Undifferentiated group.

Figure 3 illustrates the Kaplan-Meier survival curves with 95% confidence intervals for all 13 sites analyzed. Comparisons are made against the one or two most common undifferentiated histologically defined subtypes. Except for nasopharyngeal and salivary gland cancers, undifferentiated carcinomas have statistically worse overall survival compared to undifferentiated cancers with ascertained histology.

### 3.2. Demographic and Mortality Regression Data by Site

Table 1 illustrates demographic data for each of the analyzed sites, comparing undifferentiated carcinomas to the one or two most common undifferentiated histologically defined subtypes, and Table 2 presents univariate and multivariable regression data for mortality, age, sex, stage at detection, and use of treatment modalities (surgery, chemotherapy, radiotherapy).

Undifferentiated carcinomas of the nasopharynx have statistically identical demographics compared to undifferentiated squamous carcinomas (Table 1), and is the only site where these tumors have better survival compared to their histologically defined comparator, with a hazard ratio (HR) of 0.6 (95% confidence interval (CI) 0.5–0.8) (Table 2). For salivary gland tumors, undifferentiated carcinomas tend to reflect a sex distribution similar to undifferentiated mucoepidermoid carcinomas (66.7% vs. 64.3% male), compared to 79.3% male for undifferentiated squamous cell carcinomas. On both univariate and multivariable analyses, survival for undifferentiated carcinomas is identical to both mucoepidermoid carcinomas and squamous cell carcinomas (Table 2).

Compared to undifferentiated/anaplastic papillary thyroid carcinomas, undifferentiated thyroid carcinomas present at an older age (70.7 vs. 66.2 years), and overwhelmingly present as distant disease (62.5% vs. 51.2%). This likely explains why these patients are less likely to get surgery (51.9% vs. 82.6%). Median survival for undifferentiated papillary adenocarcinomas is about 10 months, versus about 3 months for undifferentiated carcinomas (Table 1). The multivariable-adjusted mortality HR compared to undifferentiated papillary thyroid carcinoma is 1.7 (1.4–1.9) (Table 2).

The most common undifferentiated cancers of the digestive system occur in the esophagus, stomach, colon/rectum, and pancreas. Demographically, undifferentiated carcinomas of the esophagus tend to resemble undifferentiated squamous carcinomas over adenocarcinomas (69.0 vs. 67.8 vs. 66.8 years, respectively) (68.5% vs. 66.3% vs. 83.8% males, respectively). Rates of unstaged disease presentation are also similar between the two groups (25.2% vs. 23.1%) (Table 1). However, survival is worse for undifferentiated esophageal carcinomas (HR 1.6 (1.3–2.0)) compared to adenocarcinomas, and to squamous cell carcinomas with HR 1.4 (1.2–1.7) (Table 2). For gastric cancers, apart from higher rates of distant disease at detection (46.7% vs. 37.3%), there are no significant differences between undifferentiated carcinomas and undifferentiated adenocarcinomas (Table 1). This is represented in the higher mortality HR compared to adenocarcinomas at 1.3 (1.1–1.4) (Table 2). For colorectal cancers, compared to undifferentiated adenocarcinomas, patients with undifferentiated carcinomas tend to be slightly older (70.4 vs. 69.0 years), female (59.3% vs. 56.2%), and substantially higher rates of distant disease at presentation (44.9% vs. 27.7%) (Table 1). Multivariable-adjusted mortality HR is 1.5 (1.4–1.7) (Table 2). For pancreatic cancers, undifferentiated carcinomas occur more often in males compared to undifferentiated adenocarcinomas (63.2% vs. 53.0%). Despite nearly similar rates of distant disease (64.8% vs. 61.7%), undifferentiated carcinoma pancreatic patients have higher rates of surgery compared to undifferentiated adenocarcinomas (57.0% vs. 43.0%) (Table 1). On univariate analysis, the mortality HR is increased for undifferentiated carcinomas at 1.14 (1.02–1.28), but loses statistical significance on multivariable analysis (1.09 (0.97–1.23)) (Table 2). Overall, across digestive system cancers, undifferentiated carcinomas have a median survival time of about 2.7–6.4 months, as opposed to about 5–12 months for undifferentiated adenocarcinomas (Table 1). Strikingly for colorectal cancer, median survival is about 6 months for undifferentiated carcinomas, but over 5 years for undifferentiated adenocarcinomas (Table 1).

Consistent with most sites, distant disease is the most likely presentation for uterine undifferentiated carcinomas at 38.1%, compared to 16.2% for undifferentiated endometroid carcinomas and 21.7% for undifferentiated adenocarcinomas. Median survival for undifferentiated carcinomas is about 16 months, compared to about 3 years for adenocarcinomas and over 5 years for endometroid carcinomas (Table 1). Mortality HR for undifferentiated uterine carcinomas to endometroid carcinomas is 1.8 (1.5–2.0), and 1.4 (1.2–1.6) to adenocarcinomas (Table 2). For ovarian cancer, there are no clinically relevant differences between undifferentiated carcinomas and cystadenocarcinomas, apart from decreased median survival (~43 vs. 52 months) (Table 1). Multivariable HR for mortality for undifferentiated carcinomas is 1.2 (1.1–1.4) relative to cystadenocarcinomas (Table 2). In breast cancer patients, undifferentiated carcinomas are similar to undifferentiated lobular carcinomas with a mean age of onset out 61 years, compared to 58.1 years for undifferentiated ductal carcinomas. Distant disease at time of diagnosis is highest for undifferentiated carcinomas at 14.7%, versus 6.5% and 5.7% for ductal and lobular carcinomas, respectively (Table 1). Mortality HR for undifferentiated carcinomas is 1.17 (1.04–1.32) relative to undifferentiated ductal carcinomas, and 1.3 (1.1–1.5) for undifferentiated lobular carcinomas (Table 2).

Similar to esophageal carcinomas, undifferentiated carcinomas of the lung have similar demographic features to undifferentiated squamous carcinomas than adenocarcinomas. For undifferentiated and squamous carcinomas, about 60% of patients are male with an average age of about 67–68 years, compared to 54% male and age 66 years for adenocarcinomas. Median survival time is about 6 months for undifferentiated carcinomas, compared to about 11–12 months for undifferentiated adenocarcinomas and squamous cell carcinomas (Table 1). Mortality HR for undifferentiated lung carcinoma is 1.2 (1.2–1.4) compared to lung adenocarcinoma, and 1.26 (1.18–1.33) to squamous cell carcinoma (Table 2). For urinary bladder cancers, undifferentiated carcinomas are more frequent in females at 34.8% compared to 20–25% for undifferentiated papillary transitional cell and transitional cell carcinomas, with a much higher rate of distant disease at 23.2%, compared to 3.1% or 10.3% for the other two types, respectively. Median survival for undifferentiated carcinoma is 8.5 months, versus well over four years for the other two histologies (Table 1). This is reflected in the multivariable mortality HR of 1.7 (1.4–2.0) to undifferentiated papillary transitional cell carcinomas, and 1.3 (1.2–1.6) to undifferentiated transitional cell carcinomas (Table 2).

Finally, we compare undifferentiated carcinomas of unknown primaries to adenocarcinomas and small cell carcinomas. For undifferentiated carcinomas, age of onset is slightly younger at 66.3 years compared to 67.0 years and 68.5 years for the other two types. There are no differences in sex distribution. Outcomes are dismal for all three cancer groups, with median survival time of 1.9 months for adenocarcinomas, 2 months for small cell carcinomas, and about 3 months for undifferentiated carcinomas (Table 1). Multivariable mortality HR is 0.85 (0.74–0.98) for undifferentiated carcinomas compared to undifferentiated adenocarcinomas, and 1.3 (1.1–1.5) to small cell carcinomas (Table 2).

## 4. Discussion

For most anatomical sites, undifferentiated carcinomas have distinct prognostic profiles independent of common demographic, clinical, and treatment parameters, when compared to undifferentiated carcinomas with an identifiable histology. Because these cancers comprise only about 0.2% of all carcinomas, the literature on them is exceedingly sparse, consisting typically of case reports. Generally, these case reports discuss a patient with distant disease, aggressive biology, indeterminate immunohistochemical staining patterns, and rapid decline despite use of conventional surgical, chemotherapeutic, and radiotherapeutic treatments. This paper represents the first systematic characterization of this disease entity by using over 40 years of population-level cancer registry data.

Undifferentiated carcinomas are characterized by early dissemination with an aggressive clinical course and a dismal prognosis [11]. The incidence rate has decreased from about 40 cases per million in 1980 to about 4 cases per million in 2017 (Figure 2). This is likely attributable to improved immunohistochemical and molecular profile techniques [12]. The era of molecular targeting small molecule inhibitors began in 2001 with the approval of the first tyrosine kinase inhibitor imatinib, and today there are nearly 100 such compounds approved for clinical use [13,14,15]. This, in part, explains the increasing divergence of incidence and mortality curves for histologically ascertainable undifferentiated carcinomas since about 2002 (Figure 2). However, this survival advantage has not been conferred to cancers that remain truly histologically undefinable, even despite better profiling tools. Such tumors have high expression of cancer stem cell-like genes, which lead to high tumorigenicity characteristics and resistance to multi-modal treatments [16].

The primary finding of this paper is that for most anatomical sites, undifferentiated carcinomas have worse prognosis compared to histologically determinable undifferentiated carcinomas, even after multivariable adjustment. There is significant variability across the sites, with thyroid cancer, colorectal cancer, and urinary bladder cancer having the largest differences in overall survival. Common mutations in genes such as *BRAF*, *TP53*, and *KRAS/NRAS* are correlated strongly with increased pathogenesis within these cancer groups, but it is not known what genetic signatures may be responsible for distinguishing these differing classes of undifferentiated cancers within each site [17,18,19]. More likely, the genetic landscape of such tumors is more plastic and pleomorphic than more conventional and better differentiated carcinomas [20]. As such, directly targeting these intrinsically therapy resistant tumors might have limited efficacy [21]. Treatment of these tumors may require whole genome sequencing for a survey of the complete mutational landscape, and clinicians could then creatively select multimodal targeted treatments along with conventional chemotherapies as toxicities would allow. Alternatively, emerging progress with immunotherapy might be one of the better treatments to improve the rather dismal prognosis of this cancer phenotype [22]. Immunotherapy has the advantage of avoiding the pitfalls of targeted therapy failure secondary to polymorphic mutational status. However, these tumors by their very nature of undifferentiation tend to acquire sufficient stemness qualities that they are more likely to be immunogenically quiescent [23]. Greater understanding of immune evasion and subsequent pharmacokinetic manipulation is probably the only viable treatment strategy for most of these tumors as they are most often metastatic at the time of diagnosis. Improved survival rates will for certain require better medical management strategies of the systemic disease side of these cancers. Patients with such cancers should be promptly referred to academic cancer centers and be highly considered for enrolment in clinical trials.

This paper does have several limitations inherent to the study of rare cancers. As a retrospective analysis, the results are prone to selection bias, and treatments are unfortunately encoded as binary variables. Broad definitions of tumor stage are also necessary to make analogous comparisons among all the sites included over four decades of data. However, no other resource outside of a population-level database can provide this level of epidemiological data. Consequently, the principal strength of using a population-level database like SEER has enabled us to systematically analyze one of the largest cohorts of undifferentiated carcinomas using a validated cancer registry well regarded for its quality and meticulous methodology [10].

## 5. Conclusions

This study provides a standardized and systematic overview of the overall demographic and histopathological features of undifferentiated carcinomas across all major sites, with direct comparisons to undifferentiated but histologically definable carcinomas within these sites. This characterization should serve as a reference to future insights into tumor biology research for these extremely aggressive and essentially orphaned tumor pathologies. Improving patient outcomes will most certainly require creatively tailored and dynamic therapies following next generation tumor molecular profiling techniques.

## Figures and Tables

**Table 1:** Baseline demographics and clinical characteristics by histology for included sites.

Site		Nasopharyngeal			Salivary Gland				Thyroid		
		Squamous Ca	Undiff. Ca	*p*-Value	Mucoepi. Ca	Squamous Ca	Undiff. Ca	*p*-Value	Pap. Adeno. Ca	Undiff. Ca	*p*-Value
**N**	**-**	97	288	-	666	111	258	-	293	1879	-
**Age Group (Years) (%)**	**0–14**	1 (1.0)	3 (1.0)	0.96	9 (1.4)	0 (0)	1 (0.4)	0.004	0 (0)	0 (0)	<0.001
	**15–29**	7 (7.2)	18 (6.2)		22 (3.3)	2 (1.8)	2 (0.8)		8 (2.7)	3 (0.2)	
	**30–49**	33 (34.0)	111 (38.5)		88 (13.2)	7 (6.3)	37 (14.3)		36 (12.3)	88 (4.7)	
	**50–65**	40 (41.2)	117 (40.6)		247 (37.1)	31 (27.9)	82 (31.8)		109 (37.2)	735 (39.1)	
	**70–85**	15 (15.5)	36 (12.5)		233 (35.0)	50 (45.0)	106 (41.1)		111 (37.9)	795 (42.3)	
	**>85**	1 (1.0)	3 (1.0)		67 (10.1)	21 (18.9)	30 (11.6)		29 (9.9)	258 (13.7)	
**Mean (SD)**	**-**	52.3 (16.1)	51.4 (15.6)	0.65	64.3 (17.7)	70.8 (15.4)	68.0 (15.4)	<0.001	66.2 (15.6)	70.7 (12.4)	<0.001
**Sex (%)**	**Male**	72 (74.2)	198 (68.8)	0.31	444 (66.7)	88 (79.3)	166 (64.3)	0.015	107 (36.5)	702 (37.4)	0.78
	**Female**	25 (25.8)	90 (31.2)		222 (33.3)	23 (20.7)	92 (35.7)		186 (63.5)	1177 (62.6)	
**Race**	**White**	42 (43.3)	98 (34.0)	0.12	555 (83.3)	99 (89.2)	227 (88.0)	0.14	247 (84.3)	1525 (81.2)	0.21
	**Black**	10 (10.3)	22 (7.6)		59 (8.9)	5 (4.5)	12 (4.7)		12 (4.1)	127 (6.8)	
	**Other**	45 (46.4)	168 (58.3)		52 (7.8)	7 (6.3)	19 (7.4)		34 (11.6)	227 (12.1)	
**Detection Stage**	**Localized**	9 (9.3)	27 (9.4)	0.69	199 (29.9)	21 (18.9)	95 (36.8)	<0.001	32 (10.9)	132 (7.0)	<0.001
	**Regional**	68 (70.1)	185 (64.2)		316 (47.4)	58 (52.3)	99 (38.4)		107 (36.5)	463 (24.6)	
	**Distant**	15 (15.5)	54 (18.8)		143 (21.5)	27 (24.3)	48 (18.6)		150 (51.2)	1174 (62.5)	
	**Unstaged**	5 (5.2)	22 (7.6)		8 (1.2)	5 (4.5)	16 (6.2)		4 (1.4)	110 (5.9)	
**Surgery**	**Yes**	84 (86.6)	250 (86.8)	0.96	634 (95.2)	99 (89.2)	235 (91.1)	0.01	242 (82.6)	975 (51.9)	<0.001
	**No**	13 (13.4)	38 (13.2)		32 (4.8)	12 (10.8)	23 (8.9)		51 (17.4)	904 (48.1)	
**Chemotherapy**	**Yes**	37 (38.1)	109 (37.8)	0.96	77 (11.6)	20 (18.0)	23 (8.9)	0.04	93 (31.7)	724 (38.5)	0.03
	**No**	60 (61.9)	179 (62.2)		589 (88.4)	91 (82.0)	235 (91.1)		200 (68.3)	1155 (61.5)	
**Radiotherapy**	**Yes**	90 (92.8)	268 (93.1)	0.93	524 (78.7)	75 (67.6)	176 (68.2)	<0.001	181 (61.8)	1068 (56.8)	0.11
	**No**	7 (7.2)	20 (6.9)		142 (21.3)	36 (32.4)	82 (1.8)		112 (38.2)	811 (43.2)	
**CSS %**	**1-year**	92 (90–93)	93 (90–95)	-	87 (83–90)	87 (75–93)	91 (84–95)	-	46 (39–52)	17 (15–19)	-
**(95% CI)**	**2-year**	85 (83–87)	85 (82–88)		76 (71–80)	70 (56–80)	81 (72–87)		39 (33–46)	11 (9–13)	
	**5-year**	74 (71–77)	75 (71–79)		62 (56–67)	50 (35–64)	64 (55–73)		34 (28–40)	8 (7–10)	
**Median**	**Months**	-	-		-	-	-		10.5	3.3	
**RS %**	**1-year**	92 (89–94)	91 (89–93)	-	87 (83–90)	79 (65–87)	90 (82–95)	-	45 (38–51)	17 (15–19)	-
**(95% CI)**	**2-year**	84 (81–87)	83 (81–86)		75 (69–80)	61 (46–73)	80 (70–87)		39 (32–45)	11 (9–12)	
	**5-year**	73 (69–77)	72 (69–75)		54 (48–60)	47 (30–62)	63 (52–72)		29 (27–41)	8 (6–10)	
**Median**	**Months**	-	-		59	47	-		10.1	3.1	
**Site**		**Esophageal**				**Gastric**			**Colorectal**		
		**Adeno. Ca**	**Squamous Ca**	**Undiff. Ca**	***p*-value**	**Adeno. Ca**	**Undiff. Ca**	***p*-Value**	**Adeno. Ca**	**Undiff. Ca**	***p*-Value**
**N**	**-**	519	416	238	-	1923	465	-	8025	594	-
**Age Group (Years) (%)**	**0–14**	0 (0)	0 (0)	0 (0)	0.01	0 (0)	0 (0)	0.67	0 (0)	0 (0)	0.09
	**15–29**	1 (0.2)	0 (0)	2 (0.8)		15 (0.8)	1 (0.2)		54 (0.7)	3 (0.5)	
	**30–49**	42 (8.1)	19 (4.6)	11 (4.6)		169 (8.8)	45 (9.7)		744 (9.3)	49 (8.2)	
	**50–65**	253 (48.7)	208 (50.0)	109 (45.8)		787 (40.9)	193 (41.5)		2956 (36.8)	190 (32.0)	
	**70–85**	188 (36.2)	165 (39.7)	88 (37.0)		794 (41.3)	186 (40.0)		3217 (40.1)	265 (44.6)	
	**>85**	35 (6.7)	24 (5.8)	28 (11.8)		158 (8.2)	40 (8.6)		1054 (13.1)	87 (14.6)	
**Mean (SD)**	**-**	66.8 (11.9)	67.8 (10.9)	69.0 (12.6)	0.06	67.7 (13.3)	67.9 (13.1)	0.75	69.0 (14.1)	70.4 (13.9)	0.02
**Sex (%)**	**Male**	435 (83.8)	276 (66.3)	163 (68.5)	<0.001	1235 (64.2)	292 (62.8)	0.57	3516 (43.8)	242 (40.7)	0.15
	**Female**	84 (16.2)	140 (33.7)	75 (31.5)		688 (35.8)	173 (37.2)		4509 (56.2)	352 (59.3)	
**Race**	**White**	502 (96.7)	324 (77.9)	201 (84.5)	<0.001	1498 (77.9)	371 (79.8)	0.21	6980 (87.0)	537 (90.4)	0.05
	**Black**	4 (0.8)	61 (14.7)	24 (10.1)		166 (8.6)	45 (9.7)		593 (7.4)	30 (5.1)	
	**Other**	13 (2.5)	31 (7.5)	13 (5.5)		259 (13.5)	49 (10.5)		452 (5.6)	27 (4.5)	
**Detection** **Stage**	**Localized**	102 (19.7)	86 (20.7)	36 (15.1)	<0.001	266 (13.8)	54 (11.6)	<0.001	1478 (18.4)	80 (13.5)	<0.001
	**Regional**	175 (33.7)	107 (25.7)	52 (21.8)		803 (41.8)	138 (29.7)		4209 (52.4)	222 (37.4)	
	**Distant**	207 (39.9)	127 (30.5)	90 (37.8)		718 (37.3)	217 (46.7)		2222 (27.7)	267 (44.9)	
	**Unstaged**	35 (6.7)	96 (23.1)	60 (25.2)		136 (7.1)	56 (12.0)		116 (1.4)	25 (4.2)	
**Surgery**	**Yes**	257 (49.5)	257 (61.8)	144 (60.5)	<0.001	1477 (76.8)	362 (77.8)	0.63	7615 (94.9)	542 (91.2)	<0.001
	**No**	262 (50.5)	159 (38.2)	94 (39.5)		446 (23.2)	103 (22.2)		410 (5.1)	52 (8.8)	
**Chemotherapy**	**Yes**	279 (53.8)	160 (38.5)	80 (33.6)	<0.001	716 (37.2)	149 (32.0)	0.04	3410 (42.5)	153 (25.8)	<0.001
	**No**	240 (46.2)	256 (61.5)	158 (66.4)		1207 (62.8)	316 (68.0)		4615 (57.5)	441 (64.2)	
**Radiotherapy**	**Yes**	247 (47.6)	253 (60.8)	129 (54.2)	<0.001	369 (19.2)	76 (16.3)	0.16	739 (9.2)	60 (10.1)	0.47
	**No**	272 (52.4)	163 (39.2)	109 (45.8)		1554 (80.8)	389 (83.7)		7286 (90.8)	534 (89.9)	
**CSS %**	**1-year**	50 (44–56)	45 (35–53)	20 (12–30)	-	50 (46–54)	37 (28–46)	-	75 (74–77)	43 (35–50)	-
**(95% CI)**	**2-year**	32 (27–38)	29 (21–38)	14 (7–23)		34 (30–38)	27 (19–35)		65 (63–66)	36 (29–43)	
	**5-year**	20 (15–25)	18 (12–26)	7 (2–15)		22 (19–26)	19 (12–27)		52 (51–54)	32 (25–39)	
**Median**	**Months**	12.0	8.5	3.8		12.0	5.3		-	7.4	
**RS %**	**1-year**	48 (43–54)	41 (32–50)	20 (12–31)	-	50 (46–53)	36 (27–45)	-	74 (73–75)	40 (33–47)	-
**(95% CI)**	**2-year**	31 (26–36)	28 (20–36)	12 (6–21)		34 (30–37)	25 (17–34)		64 (62–65)	35 (28–42)	
	**5-year**	19 (15–25)	16 (10–24)	6 (2–14)		22 (19–26)	18 (11–27)		52 (51–54)	30 (22–37)	
**Median**	**Months**	11.6	8.1	3.7		11.8	5.2		-	6.4	
		**Pancreatic**			**Uterine**				**Ovarian**		
		**Adeno. Ca**	**Undiff. Ca**	***p*-Value**	**Endomet. Ca**	**Adeno. Ca**	**Undiff. Ca**	***p*-Value**	**CystAdeno. Ca**	**Undiff. Ca**	***p*** **-Value**
**N**	**-**	939	758	-	1896	1169	515	-	10,241	840	-
**Age Group (Years) (%)**	**0–14**	0 (0)	0 (0)	0.40	0 (0)	0 (0)	0 (0)	<0.001	0 (0)	1 (0.1)	<0.001
	**15–29**	2 (0.2)	1 (0.1)		5 (0.3)	2 (0.2)	3 (0.6)		21 (0.2)	17 (2.0)	
	**30–49**	50 (5.3)	55 (7.3)		170 (9.0)	79 (6.8)	47 (9.1)		1371 (13.4)	137 (16.3)	
	**50–65**	472 (50.3)	361 (47.6)		1070 (56.4)	588 (50.3)	261 (50.7)		5721 (55.9)	410 (48.8)	
	**70–85**	365 (38.9)	293 (38.7)		555 (29.3)	418 (35.8)	163 (31.7)		2864 (28.0)	248 (29.5)	
	**>85**	50 (5.3)	48 (6.3)		96 (5.1)	82 (7.0)	41 (8.0)		264 (2.6)	27 (3.2)	
**Mean (SD)**	**-**	67.5 (11.1)	67.1 (11.8)	0.49	64.7 (12.1)	67.2 (11.9)	66.0 (12.8)	<0.001	62.9 (11.8)	62.0 (14.0)	0.04
**Sex (%)**	**Male**	498 (53.0)	479 (63.2)	<0.001	-	-	-	-	-	-	-
	**Female**	441 (47.0)	279 (36.8)		1896 (100)	1169 (100)	515 (100)		10,241 (100)	840 (100)	
**Race**	**White**	814 (86.7)	625 (82.5)	0.002	1555 (82.0)	1005 (86.0)	408 (79.2)	0.002	8812 (86.0)	735 (87.5)	0.34
	**Black**	81 (8.6)	65 (8.6)		190 (10.0)	90 (7.7)	50 (9.7)		582 (5.7)	38 (4.5)	
	**Other**	44 (4.7)	68 (9.0)		151 (8.0)	74 (6.3)	57 (11.1)		847 (8.3)	67 (8.0)	
**Detection** **Stage**	**Localized**	52 (5.5)	41 (5.4)	0.19	885 (46.7)	569 (48.7)	140 (27.2)	<0.001	427 (4.2)	77 (9.2)	<0.001
	**Regional**	252 (26.8)	171 (22.6)		664 (35.0)	285 (24.4)	161 (31.3)		1631 (15.9)	140 (16.7)	
	**Distant**	579 (61.7)	491 (64.8)		307 (16.2)	254 (21.7)	196 (38.1)		8112 (79.2)	605 (72.0)	
	**Unstaged**	56 (6.0)	55 (7.3)		40 (2.1)	61 (5.2)	18 (3.5)		71 (0.7)	18 (2.1)	
**Surgery**	**Yes**	461 (49.1)	432 (57.0)	0.001	1726 (91.0)	1030 (88.1)	448 (87.0)	0.005	9802 (95.7)	790 (94.0)	0.02
	**No**	478 (50.9)	326 (43.0)		170 (9.0)	139 (11.9)	67 (13.0)		439 (4.3)	50 (6.0)	
**Chemotherapy**	**Yes**	404 (43.0)	265 (35.0)	<0.001	692 (36.5)	252 (21.6)	217 (42.1)	<0.001	8835 (86.3)	575 (68.5)	<0.001
	**No**	535 (57.0)	493 (65.0)		1204 (63.5)	917 (78.4)	298 (57.9)		1406 (13.7)	265 (31.5)	
**Radiotherapy**	**Yes**	161 (17.1)	109 (14.4)	0.12	937 (49.4)	601 (51.4)	239 (46.4)	0.16	116 (1.1)	60 (7.1)	<0.001
	**No**	778 (82.9)	649 (85.6)		959 (50.6)	568 (48.6)	276 (53.6)		10,125 (98.9)	780 (92.9)	
**CSS %**	**1-year**	26 (21–30)	14 (10–19)	-	83 (81–85)	74 (69–78)	58 (52–63)	-	89.7 (89.0–90.4)	73 (67–77)	-
**(95% CI)**	**2-year**	10 (7–14)	7 (5–11)		75 (72–77)	59 (54–63)	46 (41–52)		76.8 (75.8–77.7)	63 (57–68)	
	**5-year**	4 (3–7)	4 (2–7)		62 (60–65)	48 (43–53)	40 (35–46)		44 (43–46)	44 (38–49)	
**Median**	**Months**	5.2	2.8		-	39.1	16.8		51.8	43.6	
**RS %**	**1-year**	25 (21–30)	15 (11–19)	-	82 (80–84)	71 (67–76)	57 (51–62)	-	90.0 (89.2–90.6)	72 (67–77)	-
**(95% CI)**	**2-year**	10 (7–13)	8 (5–11)		72 (70–75)	57 (52–62)	46 (40–51)		77.5 (76.5–78.4)	60 (56–67)	
	**5-year**	4 (2–6)	4 (2–7)		61 (58–64)	44 (39–49)	39 (33–45)		42 (44–47)	43 (37–48)	
**Median**	**Months**	5.0	2.7		-	32.7	16.0		52.8	42.9	
		**Breast**					**Lung**				
		**Ductal**	**Lobular**	**Undiff. Ca**		***p*-Value**	**Adeno. Ca**	**Squamous Ca**		**Undiff. Ca**	***p*-Value**
**N**	**-**	12,563	1787	796		-	2810	2054		2727	-
**Age Group (Years) (%)**	**0–14**	0 (0)	0 (0)	0 (0)		<0.001	0 (0)	0 (0)		1 (<0.1)	<0.001
	**15–29**	148 (1.2)	8 (0.4)	10 (1.3)			4 (0.1)	0 (0)		2 (0.1)	
	**30–49**	3739 (29.8)	417 (23.3)	183 (23.0)			229 (8.1)	82 (4.0)		204 (7.5)	
	**50–65**	5636 (44.9)	814 (45.6)	356 (44.7)			1433 (51.0)	939 (45.7)		1235 (45.3)	
	**70–85**	2598 (20.7)	466 (26.1)	200 (25.1)			1043 (37.1)	941 (45.8)		1157 (42.4)	
	**>85**	442 (3.5)	82 (4.6)	47 (5.9)			101 (3.6)	92 (4.5)		128 (4.7)	
**Mean (SD)**	**-**	58.1 (14.5)	61.1 (14.0)	61.0 (15.0)		<0.001	66.2 (11.3)	68.8 (10.1)		67.3 (11.4)	<0.001
**Sex (%)**	**Male**	75 (0.6)	5 (0.3)	4 (0.5)		0.24	1520 (54.1)	1277 (62.2)		1644 (60.3)	<0.001
	**Female**	12,488 (99.4)	1782 (99.7)	792 (99.5)			1290 (45.9)	777 (37.8)		1083 (39.7)	
**Race**	**White**	10,450 (83.2)	1589 (88.9)	670 (84.2)		<0.001	2397 (85.3)	1790 (87.1)		2241 (82.2)	<0.001
	**Black**	1174 (9.3)	120 (6.7)	86 (10.8)			241 (8.6)	172 (8.4)		283 (10.4)	
	**Other**	939 (7.5)	78 (4.4)	40 (5.0)			172 (6.1)	92 (4.5)		203 (7.4)	
**Detection Stage**	**Localized**	6789 (53.9)	932 (52.2)	326 (41.0)		<0.001	411 (14.6)	397 (19.3)		324 (11.9)	<0.001
	**Regional**	4834 (38.5)	737 (41.2)	327 (41.1)			777 (27.7)	743 (36.2)		654 (24.0)	
	**Distant**	816 (6.5)	102 (5.7)	117 (14.7)			1526 (54.3)	834 (40.6)		1626 (59.6)	
	**Unstaged**	144 (1.1)	16 (0.9)	26 (3.3)			96 (3.4)	80 (3.9)		123 (4.5)	
**Surgery**	**Yes**	12,043 (95.9)	1743 (97.5)	736 (92.5)		<0.001	1276 (45.4)	1035 (50.4)		1234 (45.3)	<0.001
	**No**	520 (4.1)	44 (2.5)	60 (7.5)			1534 (54.6)	1019 (49.6)		1493 (54.7)	
**Chemotherapy**	**Yes**	5698 (45.4)	700 (39.2)	389 (48.9)		<0.001	1179 (42.0)	689 (33.5)		1002 (36.7)	<0.001
	**No**	6865 (54.6)	1087 (60.8)	407 (51.1)			1631 (58.0)	1365 (66.5)		1725 (63.3)	
**Radiotherapy**	**Yes**	4818 (38.4)	627 (35.1)	310 (38.9)		0.03	1213 (43.2)	942 (45.9)		1158 (42.5)	0.05
	**No**	7745 (61.6)	1160 (64.9)	486 (61.1)			1597 (56.8)	1112 (54.1)		1569 (57.5)	
**CSS %**	**1-year**	95.8 (95.2–96.3)	98 (96–99)	93 (90–96)		-	51 (48–53)	50 (47–53)		35 (33–38)	-
**(95% CI)**	**2-year**	90.6 (89.8–91.4)	95 (93–96)	86 (82–90)			37 (34–39)	35 (31–38)		26 (24–29)	
	**5-year**	80.8 (79.7–81.9)	85 (82–87)	77 (71–81)			24 (22–27)	23 (21–26)		19 (17–22)	
**Median**	**Months**	-	-	-			12.5	12.0		6.7	
**RS %**	**1-year**	95.9 (95.3–96.5)	98 (96–99)	93 (89–96)		-	50 (47–52)	47 (44–50)		34 (31–36)	-
**(95% CI)**	**2-year**	91.0 (90.1–91.8)	96 (93–97)	86 (81–89)			35 (33–38)	32 (29–35)		24 (22–27)	
	**5-year**	81.2 (79.9–82.4)	86 (82–88)	77 (71–82)			23 (21–26)	20 (17–23)		17 (15–20)	
**Median**	**Months**	-	-	-			11.8	10.7		6.0	
		**Urinary Bladder**					**Unknown Primary**				
		**Pap. Trans. Ca**	**Trans. Cell Ca**	**Undiff. Ca**		***p*-Value**	**Adeno. Ca**	**Small Cell Ca**		**Undiff. Ca**	***p*-Value**
**N**	**-**	47,662	37,156	276		-	346	426		1658	-
**Age Group (Years) (%)**	**0–14**	1 (<0.1)	0 (0)	0 (0)		0.07	0 (0)	1 (0.2)		2 (0.1)	0.09
	**15–29**	31 (0.1)	14 (<0.1)	0 (0)			2 (0.6)	3 (0.7)		28 (1.7)	
	**30–49**	1304 (2.7)	1078 (2.9)	9 (3.3)			41 (11.8)	30 (7.0)		167 (10.1)	
	**50–65**	16,442 (34.5)	12,830 (34.5)	85 (30.8)			145 (41.9)	173 (40.6)		709 (42.8)	
	**70–85**	22,763 (47.8)	17,439 (46.9)	143 (51.8)			125 (36.1)	179 (42.0)		637 (38.4)	
	**>85**	7121 (14.9)	5795 (15.6)	39 (14.1)			33 (9.5)	40 (9.4)		115 (6.9)	
**Mean (SD)**	**-**	72.6 (11.3)	72.7 (11.4)	73.0 (11.6)		0.70	67.0 (13.9)	68.5 (13.1)		66.3 (14.1)	0.01
**Sex (%)**	**Male**	38,019 (79.8)	27,659 (74.4)	180 (65.2)		<0.001	172 (49.7)	234 (54.9)		892 (53.8)	0.30
	**Female**	9643 (20.2)	9497 (25.6)	96 (34.8)			174 (50.3)	192 (45.1)		766 (46.2)	
**Race**	**White**	42,870 (89.9)	33,020 (88.9)	242 (87.7)		<0.001	308 (89.0)	386 (90.6)		1433 (86.4)	0.13
	**Black**	2245 (4.7)	2261 (6.1)	25 (9.1)			22 (6.4)	26 (6.1)		126 (7.6)	
	**Other**	2547 (5.3)	1875 (5.0)	9 (3.3)			16 (4.6)	14 (3.3)		99 (6.0)	
**Detection Stage**	**In Situ**	14,924 (31.3)	2162 (5.8)	0 (0)		<0.001	-	-		-	-
	**Localized**	26,789 (56.2)	20,495 (55.2)	75 (27.2)			-	-		-	
	**Regional**	4084 (8.6)	9827 (26.7)	107 (38.8)			-	-		-	
	**Distant**	1461 (3.1)	3821 (10.3)	64 (23.2)			-	-		-	
	**Unstaged**	404 (0.8)	851 (2.3)	30 (10.9)			346 (100)	426 (100)		1658 (100)	
**Surgery**	**Yes**	46,405 (97.4)	35,218 (94.8)	254 (92.0)		<0.001	288 (83.2)	318 (74.6)		1511 (91.1)	<0.001
	**No**	1257 (2.6)	1938 (5.2)	22 (8.0)			58 (16.8)	108 (25.4)		147 (8.9)	
**Chemotherapy**	**Yes**	11,885 (24.9)	11,670 (31.4)	55 (19.9)		<0.001	131 (37.9)	160 (37.6)		437 (26.4)	<0.001
	**No**	35,777 (75.1)	25,486 (68.6)	221 (80.1)			215 (62.1)	266 (62.4)		1221 (73.6)	
**Radiotherapy**	**Yes**	2544 (5.3)	5390 (14.5)	96 (34.8)		<0.001	95 (27.5)	98 (23.0)		552 (33.3)	<0.001
	**No**	45,118 (94.7)	31,766 (85.5)	180 (65.2)			251 (72.5)	328 (77.0)		1106 (66.7)	
**CSS %**	**1-year**	92.1 (91.8–92.4)	75.2 (74.6–75.8)	42 (28–56)		-	20 (12–30)	22 (16–28)		28 (23–32)	-
**(95% CI)**	**2-year**	86.4 (86.0–86.8)	62.9 (62.2–63.5)	36 (22–49)			10 (4.4–18)	13 (8.5–19)		20 (16–24)	
	**5-year**	76.7 (76.2–77.2)	51.5 (50.8–52.2)	27 (15–40)			6.7 (2.3–14)	11 (6.9–17)		16 (12–20)	
**Median**	**Months**	-	-	10.6			1.9	2.1		3.5	
**RS %**	**1-year**	91.8 (91.4–92.2)	72.8 (72.2–73.5)	38 (25–52)		-	18 (11–27)	20 (14–26)		25 (21–29)	-
**(95% CI)**	**2-year**	86.0 (85.5–86.4)	60.2 (59.5–60.9)	33 (20–47)			7.5 (3.1–15)	12 (7.9–18)		17 (14–21)	
	**5-year**	75.9 (75.2–76.6)	48.4 (47.6–49.2)	27 (15–40)			5.2 (1.7–12)	9.8 (5.9–15)		13 (10–17)	
**Median**	**Months**	-	52.4	8.5			1.8	1.9		2.8	

Squamous Ca, squamous cell carcinoma; Adeno. Ca, adenocarcinoma; Undiff. Ca, undifferentiated carcinoma; Mucoepi. Ca, mucoepidermoid carcinoma; Pap. Adeno. Ca, papillary adenocarcinoma; Endomet. Ca, endometroid carcinoma; CystAdeno. Ca, cystadenocarcinoma; Pap. Trans. Ca, papillary transitional cell carcinoma; Trans. Cell Ca, transitional cell carcinoma; CSS, cause-specific survival; RS, relative survival.

**Table 2:** Derived univariate and multivariable Cox-proportional hazard ratios (HR) of mortality for included sites.

Site	Nasopharyngeal		Salivary Gland				Thyroid	
	Carcinoma Undiff. vs. Squamous Cell Undiff.		Carcinoma Undiff. vs. Mucoepidermoid Carcinoma Undiff.		Carcinoma Undiff. vs. Squamous Cell Undiff.		Carcinoma Undiff. vs. Papillary Adeno Ca Undiff.	
HR (95% CI)	Univariate	Multivariable	Univariate	Multivariable	Univariate	Multivariable	Univariate	Multivariable
**Undiff. Histology**	0.7 (0.5–0.9)	0.6 (0.4–0.8)	1.1 (0.9–1.4) *	1.1 (0.9–1.4) *	1.0 (0.7–1.4) *	1.3 (0.9–1.9) *	2.0 (1.7–2.3)	1.7 (1.4–1.9)
**Age (per 10 years)**	1.3 (1.1–1.4)	1.3 (1.2–1.5)	1.23 (1.15–1.32)	1.24 (1.15–1.33)	1.1 (1.0–1.3)	1.2 (1.1–1.3)	1.25 (1.20–1.30)	1.2 (1.1–1.3)
**Sex (Female)**	1.0 (0.7–1.3) *	0.9 (0.6–1.2) *	0.8 (0.6–1.0)	0.8 (0.7–1.0) *	0.9 (0.6–1.3) *	0.9 (0.6–1.3) *	1.0 (0.9–1.1) *	1.0 (0.9–1.1) *
**Race**								
**Black**	0.9 (0.5–1.5) *	1.2 (0.7–2.0) *	0.6 (0.4–0.9)	0.7 (0.4–1.1) *	0.9 (0.4–2.0) *	1.3 (0.5–2.9) *	1.0 (0.8–1.2) *	1.1 (0.9–1.3) *
**Other**	0.6 (0.5–0.8)	0.6 (0.4–0.8)	0.7 (0.4–1.0) *	0.6 (0.4–1.0)	0.7 (0.4–1.4) *	0.6 (0.3–1.2) *	1.1 (0.9–1.2) *	1.0 (0.9–1.2) *
**Detection Stage**								
**Regional**	1.3 (0.8–2.3) *	1.4 (0.8–2.5) *	2.7 (1.7–3.0)	2.3 (1.7–3.0)	2.1 (1.3–3.2)	2.1 (1.4–3.3)	1.9 (1.5–2.5)	2.1 (1.6–2.7)
**Distant**	3.8 (2.1–6.8)	5.1 (2.8–9.3)	4.3 (3.2–5.8)	3.7 (2.7–5.1)	4.3 (2.7–6.8)	3.7 (2.2–6.0)	3.6 (2.8–4.5)	3.5 (2.8–4.5)
**Unstaged**	1.5 (0.7–3.1)	1.4 (0.7–3.0) *	3.3 (1.8–6.1)	2.2 (1.1–4.1)	2.5 (1.2–5.2)	2.2 (1.0–4.5)	2.7 (2.0–3.7)	1.8 (1.3–2.5)
**Surgery (Yes)**	0.9 (0.6–1.4) *	1.0 (0.7–1.6) *	0.4 (0.2–0.5)	0.5 (0.3–0.7)	0.6 (0.4–0.9)	0.7 (0.4–1.2) *	0.49 (0.44–0.54)	0.63 (0.57–0.71)
**Chemotherapy (Yes)**	1.2 (0.9–1.6) *	1.3 (0.92–1.71) *	2.1 (1.6–2.8)	1.7 (1.3–2.3)	2.3 (1.5–3.4)	2.0 (1.2–3.2)	0.9 (0.8–1.0) *	0.88 (0.78–0.98)
**Radiotherapy (Yes)**	0.5 (0.3–0.8)	0.6 (0.3–1.0)	0.9 (0.7–1.1) *	0.8 (0.6–1.0) *	1.2 (0.8–1.7) *	1.0 (0.7–1.4) *	0.73 (0.66–0.81)	0.7 (0.6–0.8)
**Site**	**Esophageal**				**Gastric**		**Colorectal**	
	**Carcinoma Undff. vs.** **Adenocarcinoma Undiff.**		**Carcinoma Undiff. vs.** **Squamous Cell Undiff.**		**Carcinoma Undff. vs.** **Adenocarcinoma Undiff.**		**Carcinoma Undff. vs.** **Adenocarcinoma Undiff.**	
**HR (95% CI)**	**Univariate**	**Multivariable**	**Univariate**	**Multivariable**	**Univariate**	**Multivariable**	**Univariate**	**Multivariable**
**Undiff. Histology**	1.7 (1.5–2.1)	1.6 (1.3–2.0)	1.5 (1.3–1.9)	1.4 (1.2–1.7)	1.3 (1.2–1.5)	1.3 (1.1–1.4)	1.8 (1.6–2.0)	1.5 (1.4–1.7)
**Age (per 10 years)**	1.03 (0.96–1.11) *	1.0 (0.9–1.1) *	1.1 (1.0–1.2) *	1.12 (1.02–1.22)	1.04 (1.01–1.09)	1.05 (1.01–1.10)	1.05 (1.02–1.07)	1.13 (1.10–1.16)
**Sex (Female)**	1.0 (0.9–1.3) *	1.0 (0.8–1.2) *	0.8 (0.7–1.0) *	0.8 (0.7–1.0) *	1.0 (0.9–1.1) *	1.0 (0.9–1.1) *	0.92 (0.86–0.98)	0.90 (0.84–0.96)
**Race**								
**Black**	1.8 (1.1–2.8)	1.2 (0.8–2.0) *	1.0 (0.7–1.3) *	1.0 (0.8–1.4) *	0.9 (0.8–1.1) *	1.0 (0.8–1.2) *	1.0 (0.9–1.2) *	1.0 (0.9–1.1) *
**Other**	1.0 (0.6–1.6) *	0.9 (0.5–1.4) *	0.6 (0.5–0.9)	0.7 (0.5–0.9)	0.7 (0.6–0.8)	0.83 (0.71–0.97)	0.91 (0.79–1.04) *	0.91 (0.79–1.04) *
**Detection Stage**								
**Regional**	1.6 (1.2–2.1)	2.0 (1.5–2.6)	1.2 (0.9–1.5) *	1.3 (1.0–1.8) *	2.0 (1.7–2.4)	2.4 (2.0–2.8)	3.0 (2.7–3.5)	3.6 (3.1–4.1)
**Distant**	3.1 (2.4–4.0)	3.3 (2.5–4.3)	2.3 (1.8–3.0)	2.6 (2.0–3.5)	5.1 (4.3–6.1)	5.6 (4.7–6.8)	12 (11–14)	16 (14–18)
**Unstaged**	2.8 (2.0–3.8)	2.3 (1.6–3.2)	1.5 (1.1–2.0)	1.4 (1.1–1.9)	3.8 (3.0–4.8)	3.5 (2.7–4.5)	7.1 (5.5–9.4)	5.5 (4.2–7.3)
**Surgery (Yes)**	0.6 (0.5–0.7)	0.7 (0.5–0.8)	0.9 (0.8–1.1) *	0.9 (0.7–1.1) *	0.54 (0.48–0.61)	0.70 (0.61–0.80)	0.32 (0.28–0.35)	0.56 (0.50–0.64)
**Chemotherapy (Yes)**	0.9 (0.8–1.0) *	0.7 (0.5–0.8)	0.6 (0.5–0.8)	0.5 (0.4–0.7)	0.94 (0.85–1.04) *	0.76 (0.68–0.86)	1.08 (1.01–1.15)	0.67 (0.63–0.72)
**Radiotherapy (Yes)**	1.0 (0.8–1.2) *	0.9 (0.8–1.1) *	0.8 (0.6–0.9)	0.9 (0.7–1.1) *	0.75 (0.66–0.85)	0.78 (0.68–0.89)	1.13 (1.03–1.25)	1.08 (0.97–1.19) *
**Site**	**Pancreatic**		**Uterine**				**Ovarian**	
	**Carcinoma Undff. vs.** **Adenocarcinoma Undiff.**		**Carcinoma Undiff. vs.** **Endometrioid Carcinoma Undiff.**		**Carcinoma Undiff. vs.** **Adenocarcinoma Undiff.**		**Carcinoma Undiff. vs.** **Cystadenocarcinoma Undiff.**	
**HR (95% CI)**	**Univariate**	**Multivariable**	**Univariate**	**Multivariable**	**Univariate**	**Multivariable**	**Univariate**	**Multivariable**
**Undiff. Histology**	1.14 (1.02–1.28)	1.09 (0.97–1.23) *	2.1 (1.8–2.5)	1.8 (1.5–2.0)	1.7 (1.4–1.9)	1.4 (1.2–1.6)	1.2 (1.1–1.3)	1.2 (1.1–1.4)
**Age (per 10 years)**	1.09 (1.03–1.14)	1.06 (1.00–1.11)	1.14 (1.08–1.21)	1.13 (1.06–1.19)	1.12 (1.05–1.19)	1.16 (1.09–1.23)	1.22 (1.19–1.25)	1.18 (1.16–1.21)
**Sex (Female)**	0.87 (0.79–0.98)	0.93 (0.83–1.04) *	-	-	-	-	-	-
**Race**								
**Black**	1.0 (0.9–1.3) *	1.0 (0.8–1.2) *	1.4 (1.1–1.7)	1.2 (1.0–1.5) *	1.6 (1.2–2.1)	1.2 (1.0–1.6) *	1.3 (1.1–1.4)	1.3 (1.1–1.4)
**Other**	1.0 (0.8–1.3) *	0.9 (0.7–1.1) *	1.0 (0.8–1.3) *	1.1 (0.8–1.3) *	1.0 (0.8–1.4) *	1.0 (0.8–1.3) *	0.85 (0.77–0.93)	0.87 (0.79–0.95)
**Detection Stage**								
**Regional**	1.2 (0.9–1.5) *	1.4 (1.1–1.8)	3.1 (2.6–3.7)	3.4 (2.8–4.1)	3.3 (2.7–4.0)	3.4 (2.7–4.1)	1.9 (1.6–2.4)	2.0 (1.6–2.5)
**Distant**	2.1 (1.6–2.7)	2.3 (1.8–3.0)	9.5 (7.9–11)	8.3 (6.7–10)	8.5 (7.0–10)	8.0 (6.5–9.8)	5.6 (4.6–6.9)	6.0 (4.9–7.3)
**Unstaged**	1.5 (1.1–2.1)	1.4 (1.0–1.9)	4.8 (3.2–7.3)	2.1 (1.4–3.3)	2.6 (1.7–3.8)	1.8 (1.2–2.7)	6.7 (4.8–9.3)	3.8 (2.7–5.3)
**Surgery (Yes)**	0.86 (0.77–0.96)	1.0 (0.9–1.1) *	0.24 (0.20–0.29)	0.37 (0.30–0.45)	0.29 (0.23–0.35)	0.48 (0.39–0.59)	0.31 (0.27–0.34)	0.39 (0.35–0.44)
**Chemotherapy (Yes)**	0.64 (0.57–0.72)	0.63 (0.56–0.72)	1.7 (1.5–1.9)	0.9 (0.8–1.0) *	1.8 (1.6–2.1)	0.9 (0.7–1.0) *	0.78 (0.73–0.84)	0.67 (0.62–0.72)
**Radiotherapy (Yes)**	0.62 (0.54–0.71)	0.80 (0.69–0.93)	0.61 (0.53–0.69)	0.67 (0.59–0.76)	0.70 (0.61–0.80)	0.77 (0.67–0.89)	1.3 (1.1–1.6)	1.3 (1.1–1.6)
**Site**	**Breast**				**Lung**			
	**Carcinoma Undff. vs.** **Ductal Undiff.**		**Carcinoma Undff. vs.** **Lobular Undiff.**		**Carcinoma Undff. vs.** **Adenocarcinoma Undiff.**		**Carcinoma Undiff. vs.** **Squamous Cell Undiff.**	
**HR (95% CI)**	**Univariate**	**Multivariable**	**Univariate**	**Multivariable**	**Univariate**	**Multivariable**	**Univariate**	**Multivariable**
**Undiff. Histology**	1.4 (1.3–1.6)	1.17 (1.04–1.32)	1.5 (1.3–1.8)	1.3 (1.1–1.5)	1.3 (1.2–1.4)	1.26 (1.18–1.33)	1.3 (1.2–1.4)	1.15 (1.07–1.23)
**Age (per 10 years)**	1.08 (1.06–1.11)	1.09 (1.06–1.12)	1.09 (1.04–1.15)	1.12 (1.07–1.19)	1.06 (1.03–1.09)	1.07 (1.04–1.10)	1.05 (1.02–1.09)	1.07 (1.03–1.10)
**Sex (Female)**	0.61 (0.44–0.86)	0.74 (0.52–1.03) *	0.9 (0.3–2.8) *	1.1 (0.3–3.3) *	0.89 (0.83–0.94)	0.85 (0.79–0.90)	0.94 (0.87–1.00) *	0.90 (0.84–0.96)
**Race**								
**Black**	1.3 (1.2–1.4)	1.2 (1.1–1.4)	1.4 (1.1–1.8)	1.4 (1.1–1.8)	1.14 (1.03–1.27)	1.01 (0.91–1.13) *	1.2 (1.1–1.3)	1.0 (0.9–1.2) *
**Other**	0.8 (0.7–0.9)	0.85 (0.75–0.97)	0.9 (0.7–1.3) *	1.0 (0.7–1.3) *	0.91 (0.80–1.03) *	0.83 (0.73–0.95)	1.1 (0.9–1.2) *	0.9 (0.8–1.1) *
**Detection Stage**								
**Regional**	2.9 (2.7–3.1)	2.9 (2.7–3.1)	3.0 (2.6–3.6)	3.2 (2.7–3.8)	1.8 (1.6–2.1)	1.9 (1.7–2.2)	1.7 (1.5–1.9)	1.7 (1.5–2.0)
**Distant**	14 (13–16)	12 (11–14)	18 (15–23)	18 (14–22)	5.0 (4.5–5.6)	5.0 (4.4–5.6)	4.8 (4.2–5.3)	4.5 (3.9–5.0)
**Unstaged**	6.2 (5.0–7.6)	4.6 (3.7–5.7)	8.2 (5.5–12)	6.8 (4.4–10)	3.5 (2.9–4.2)	3.0 (2.5–3.6)	3.1 (2.6–3.8)	2.6 (2.1–3.2)
**Surgery (Yes)**	0.20 (0.18–0.22)	0.53 (0.47–0.60)	0.20 (0.15–0.25)	0.8 (0.6–1.0) *	0.48 (0.45–0.51)	0.71 (0.66–0.76)	0.47 (0.44–0.50)	0.69 (0.64–0.75)
**Chemotherapy (Yes)**	1.34 (1.26–1.43)	1.0 (0.9–1.1) *	1.4 (1.3–1.6)	1.0 (0.8–1.1) *	1.07 (1.01–1.14)	0.69 (0.65–0.74)	1.04 (0.97–1.12) *	0.70 (0.65–0.76)
**Radiotherapy (Yes)**	0.90 (0.84–0.95)	0.87 (0.82–0.93)	1.0 (0.9–1.2) *	0.9 (0.8–1.1) *	1.5 (1.4–1.6)	1.2 (1.1–1.3)	1.5 (1.4–1.6)	1.2 (1.1–1.3)
**Site**	**Urinary Bladder**				**Unknown Primary**			
	**Carcinoma Undff. vs.** **Papillary Transition Cell Undiff.**		**Carcinoma Undiff. vs.** **Transitional Cell Undiff.**		**Carcinoma Undff. vs.** **Adenocarcinoma Undiff.**		**Carcinoma Undff. vs.** **Small Cell Carcinoma Undiff.**	
**HR (95% CI)**	**Univariate**	**Multivariable**	**Univariate**	**Multivariable**	**Univariate**	**Multivariable**	**Univariate**	**Multivariable**
**Undiff. Histology**	4.6 (3.9–5.4)	1.7 (1.4–2.0)	1.9 (1.7–2.3)	1.3 (1.2–1.6)	0.82 (0.71–0.94)	0.85 (0.74–0.98)	1.17 (1.01–1.35)	1.3 (1.1–1.5)
**Age (per 10 years)**	1.44 (1.41–1.46)	1.49 (1.46–1.52)	1.27 (1.25–1.29)	1.34 (1.32–1.36)	1.10 (1.05–1.13)	1.11 (1.07–1.16)	1.08 (1.04–1.12)	1.10 (1.06–1.15)
**Sex (Female)**	1.26 (1.20–1.31)	1.09 (1.04–1.14)	1.36 (1.32–1.41)	1.17 (1.14–1.21)	0.95 (0.86–1.06) *	0.93 (0.83–1.03) *	1.0 (0.9–1.1) *	1.0 (0.9–1.1) *
**Race**								
**Black**	1.28 (1.19–1.39)	1.34 (1.24–1.45)	1.35 (1.27–1.43)	1.3 (1.2–1.4)	1.2 (1.0–1.5)	1.3 (1.1–1.6)	1.3 (1.1–1.6)	1.3 (1.1–1.6)
**Other**	0.93 (0.86–1.01) *	0.94 (0.87–1.02) *	0.85 (0.79–0.91)	0.85 (0.79–0.92)	0.9 (0.7–1.1) *	0.9 (0.7–1.2) *	0.9 (0.7–1.2) *	0.9 (0.7–1.2) *
**Detection Stage**								
**In Situ**	0.42 (0.40–0.45)	0.43 (0.41–0.45)	0.38 (0.35–0.43)	0.39 (0.35–0.43)	-	-	-	-
**Regional**	2.8 (2.6–2.9)	2.8 (2.7–3.0)	1.78 (1.72–1.84)	1.9 (1.8–2.0)	-	-	-	-
**Distant**	9.2 (8.7–9.8)	9.5 (8.9–10)	5.1 (4.9–5.3)	5.6 (5.4–5.9)	-	-	-	-
**Unstaged**	1.7 (1.5–2.0)	1.5 (1.3–1.8)	1.5 (1.4–1.7)	1.3 (1.2–1.5)	-	-	-	-
**Surgery (Yes)**	0.76 (0.68–0.84)	0.73 (0.65–0.81)	0.72 (0.67–0.77)	0.76 (0.71–0.82)	0.9 (0.7–1.0) *	0.9 (0.7–1.1) *	0.9 (0.8–1.1) *	0.9 (0.8–1.1) *
**Chemotherapy (Yes)**	1.28 (1.23–1.33)	0.92 (0.88–0.96)	1.12 (1.09–1.16)	0.89 (0.86–0.92)	1.2 (1.1–1.3)	1.2 (1.1–1.4)	1.17 (1.04–1.31)	1.3 (1.1–1.4)
**Radiotherapy (Yes)**	3.5 (3.3–3.6)	1.7 (1.6–1.8)	1.75 (1.69–1.82)	1.33 (1.28–1.38)	0.78 (0.70–0.87)	0.8 (0.7–0.9)	0.77 (0.69–0.87)	0.81 (0.72–0.91)

*p* < 0.05 relative to reference unless noted by * *p* ≥ 0.05. Reference categories: Sex (Male), Race (White), Detection Stage (Localized), Surgery (No), Chemotherapy (No), Radiotherapy (No). CI, confidence interval. Undiff., undifferentiated.

## Data Availability

Data release from the SEER database (https://seer.cancer.gov/, accessed on 28 October 2022).

## Supplementary Materials

The following supporting information can be downloaded at: https://www.mdpi.com/article/10.3390/cancers14235819/s1, Table S1: Exclusion criteria and counts of all cases and undifferentiated carcinoma cases. Table S2: Variables in analysis. Table S3: Breakdown of undifferentiated carcinoma cases in SEER (1975–2017), both analyzed and not analyzed.
