# Supplementary material for: Epidemiology of Undifferentiated Carcinomas
Source: Cancers (Basel). 2022 Nov 25;14(23):5819. doi: 10.3390/cancers14235819 (PMC9740284; doi:10.3390/cancers14235819)
Supplement: Supplementary file 1 [file cancers-14-05819-s001.zip › cancers-2031179-supplementary.pdf]

cancers MDPI

Supplementary Material

Epidemiology of Undifferentiated Carcinomas

Table S1. Exclusion criteria and counts of all cases and undifferentiated carcinoma cases.

Data Processing Scheme	Count (All)	Count (Undifferentiated Carcinoma)
Initial count of all non-blood borne cancer cases diagnosed 1975-2017.	9,217,067	18,382
Drop if age is unknown.	9,217,067	18,382
Drop if gender is unknown.	9,217,067	18,382
Drop if race is unknown.	9,217,067	18,347
Drop if stage is uncoded.	8,102,725	12,437
Drop if surgical status is unknown.	8,002,108	12,404
Drop if chemotherapy status is unknown.	8,002,108	12,404
Drop if radiation therapy status is unknown.	8,002,108	12,404
Drop if cause-specific death classification is unknown/missing.	7,930,897	12,247
Drop if survival months are unknown/missing.	7,903,374	12,167

Table S2. Variables in analysis. Categorization reflects final variable composition.

Variable (SEER Variable)	Variable Name/Description per SEER	Categorization
**Exposure**		
Histology - ICD-0-3 (histo)	8020/3 (Carcinoma, Undifferentiated, NOS)<br>8021/3 (Carcinoma, Anaplastic, NOS)<br>8022/3 (Pleomorphic Carcinoma)	Undifferentiated Carcinoma – 802x
	814x/3 (Adenocarcinoma)<br>807x/3 (Squamous Cell Carcinoma)<br>8430/3 (Mucoepidermoid Carcinoma) (Nasopharyngeal)<br>8260/3 (Papillary Adenocarcinoma) (Thyroid)<br>838x/3 (Endometroid Carcinoma) (Uterine)<br>84(4-7)x/3 (Cystadenocarcinoma) (Ovarian)<br>850x/3 (Ductal Carcinoma) (Breast)<br>852x/3 (Lobular and other Ductal Ca) (Breast)<br>812x/3 (Papillary Transitional Cell Carcinoma) (Urinary Bladder)<br>813x/3 (Transitional Cell Carcinoma) (Urinary Bladder)<br>804x/3 (Small Cell/Non-Small Cell Carcinoma) (Unknown Primary)	Adenocarcinoma - 814x<br>Squamous Cell Carcinoma – 807x<br>Mucoepidermoid – 843x<br>Papillary Adenocarcinoma – 826x<br>Endometroid Carcinoma – 839x<br>Cystadenocarcinoma – 84(4-7)x<br>Ductal – 850x<br>Lobular – 852x<br>Papillary Transition Cell – 812x<br>Transition Cell Carcinoma – 813x<br>Small Cell Carcinoma – 804x
**Outcome**		
Mortality (seercausespecific-deathclassifica)	SEER cause-specific death classification<br>0 (Alive or dead of other cause), 1 (Dead), 8 (Dead – missing/un-known cause of death), 9 (N/A not first tumor)	Alive/N/A – 0,9<br>Dead – 1
(survivalmonths)	Survival months<br>0-9998 (in months), 9999 (Unknown)	Months (0-9998)
**Co-variables**		
Age (agerecode-withsingleage-sand100)	000-100 (Actual age of diagnosis in years), 999 (Unknown)	-
Sex (sex)	1 (Male), 2 (Female)	Male – 1<br>Female – 2
Race	1 (white), 2 (black), 3 (other), 9 (unknown)	White – 1

(racerecode-whiteblackother)		Black – 2<br>Other – 3
Detection Stage (sumarystage2000 19982017) (1998-2017) & (seerhistori-calstagea19732015) (1975-1997)	0 (In situ —A noninvasive neoplasm; a tumor which has not penetrated the basement membrane nor extended beyond the epithelial tissue)<br>1 (Localized —An invasive neoplasm confined entirely to the organ of origin. It may include intraluminal extension where specified)<br>2 (Regional —A neoplasm that has extended 1) beyond the limits of the organ of origin directly into surrounding organs or tissues; 2) into regional lymph nodes by way of the lymphatic system; or 3) by a combination of extension and regional lymph nodes)<br>4 (Distant —A neoplasm that has spread to parts of the body remote from the primary tumor either by direct extension or by discontinuous metastasis to distant organs, issues, or via the lymphatic system to distant lymph nodes)<br>9 (Unstaged —Information is not sufficient to assign a stage)	In situ – 0<br>Localized – 1<br>Regional – 2<br>Distant – 4<br>Unknown – 9
Grade Differentiation (gradethru2017)	1 (Grade I; grade i; grade 1; well differentiated; differentiated, NOS), 2 (Grade II; grade ii; grade 2; moderately differentiated; moderately differentiated; intermediate differentiation), 3 (Grade III; grade iii; grade 3; poorly differentiated; differentiated), 4 (Grade IV; grade iv; grade 4; undifferentiated; anaplastic), 5 (T-cell; T-precursor), 6 (B-cell; Pre-B; B-Precursor), 7 (Null cell; Non T-non B), 8 (NK cell (natural killer cell)), 9 (cell type not determined, not stated or not applicable)	Well – 1<br>Moderate – 2<br>Poor – 3<br>Undifferentiated – 4<br>Unknown – 9
Surgery (sitespecificsurgery19731997vari (1975-1997) & rxsummarysurg-primstie1998 (1998-2017)	00 (None, no surgical procedure of primary site, diagnosed at autopsy only), 10-98 (site specific codes), 99 (unknown if surgery performed; death certificate only)	Yes – 01-98<br>No – 00
Chemotherapy (chemotherapyreo-dodeyesnounk)	0 (None/Unknown), 1 (Yes)	Yes – 1<br>No – 0
Radiotherapy (radiationrecode)	0 (None/Unknown, diagnosed at autopsy), 1 (Beam radiation), 2 (Radioactive implants), 3 (Radioisotopes), 4 (Combination of 1 with 2 or 3), 5 (Radiation, NOS – method or source not specified), 6 (Other radiation – 1973-1987 cases only), 7 (Patient or patient’s guardian refused radiation therapy), 8 (Radiation recommended, unknown if administered)	Yes – 1,2,4,3,4,5,6<br>No – 0,7,8

Table S3. Breakdown of undifferentiated carcinoma cases in SEER (1975-2017), both analyzed and not analyzed.

Site	SEER Site Recode	Count	% Total
**Sites Analyzed**		**11,292**	**92.80**
**Head and Neck**		**2,425**	**19.93**
Nasopharyngeal	(20060) Nasopharynx	288	2.37
Salivary Gland	(20030) Salivary Gland	258	2.12
Thyroid	(32010) Thyroid	1,879	15.44
**Digestive System**		**2,055**	**16.89**
Esophagus	21010 (Esophagus)	238	1.96
Gastric	21020 (Stomach)	465	3.82
Colorectal	21041 (Cecum), 21042 (Appendix), 21043, (Ascending Colon), 21044 (Hepatic Flexure), 21045 (Transverse Colon), 21046 (Splenic Flexure), 21047 (Descending Colon), 21048 (Sigmoid Colon), 21049 (Large Intestine, NOS), 21051 (Rectosigmoid Junction), 21052 (Rectum)	594	4.88
Pancreatic	21100 (Pancreas)	758	6.23
**Female Reproductive System**		**1,355**	**11.13**
Uterine	27020 (Corpus Uteri), 20730 (Uterus, NOS)	515	4.23
Ovarian	27040 (Ovary)	840	6.90
**Breast**	26000 (Breast)	**796**	**6.54**
**Lung**	22030 (Lung and Bronchus)	**2,727**	**22.41**
**Urinary Bladder**	29010 (Urinary Bladder)	**276**	**2.27**
**Unknown Primary**	37000 (Miscellaneous)	**1658**	**13.63**
**Sites Not Analyzed**		**875**	**7.20**
Oral Cavity/Pharynx	20010 (Lip), 20020 (Tongue), 20040 (Floor of Mouth), 20050 (Gum and Other Mouth), 20070 (Tonsil), 20080 (Oropharynx), 20090 (Hypopharynx), 20100 (Other Oral Cavity and Pharynx)	135	1.11
Anus	21060 (Anus, Anal Canal and Anorectum)	20	0.16
Liver/Biliary Tree	21071 (Liver), 21072 (Intrahepatic Bile Duct), 21080 (Gallbladder), 21090 (Other Biliary)	176	1.45
Peritoneum	21110 (Retroperitoneum), 21120 (Peritoneum, Omentum and Mesentery)	5	0.04
Other Digestive	21030 (Small Intestine), 21130 (Other Digestive Organs)	73	0.60
Respiratory System	22010 (Nose, Nasal Cavity and Middle Ear), 22020 (Larynx), 22060 (Trachea, Mediastinum and Other Respiratory Organs)	129	1.06
Soft Tissue	24000 (Soft Tissue including Heart)	10	0.08
Female Genital System	27010 (Cervix Uteri), 27050 (Vagina), 27060 (Vulva), 27070 (Other Female Genital Organs)	191	1.58
Male Genital System	28010 (Prostate), Testis (28020), Penis (28040)	38	0.32
Urinary System	29020 (Kidney and Renal Pelvis), 29030 (Ureter), 29040 (Other Urinary Groups)	98	0.81
